# DAMP: bridging mechanotransduction and immune responses in tendinopathy

**DOI:** 10.3389/fimmu.2026.1910397

**Published:** 2026-10-14

**Authors:** Hongjun Zheng, Shihua Gao, Zhuangfeng Weng

**Affiliations:** 1 Shenzhen Key Laboratory of Systems Medicine for Inflammatory Diseases, Zhongshan School of Medicine, Sun Yat-sen University, Sun Yat-Sen University, Shenzhen, China; 2 Department of Orthopedics, Zhongshan Hospital of Traditional Chinese Medicine Affiliated to Guangzhou University of Traditional Chinese Medicine, Zhongshan, Guangdong, China

**Keywords:** DAMPs, inflammation, signaling pathways, tendon, treatment

## Abstract

Tendinopathy is increasingly recognized as a chronic degenerative disorder driven by sterile inflammation, where persistent mechanical stress and dysregulated innate immunity converge to disrupt tissue homeostasis. Damage-associated molecular patterns (DAMPs), released from stressed or injured tenocytes and resident immune cells, serve as key mediators translating mechanical overload into sustained inflammatory responses. Their persistent release perpetuates inflammation, extracellular matrix disorganization, and impaired tissue repair, thereby establishing a self-sustaining cycle of degeneration. Recent studies have highlighted DAMPs as central regulators in the initiation and maintenance of tendon inflammation, reflecting an intricate interplay between mechanical cues, metabolic stress, and immune dysfunction. This review summarizes current understanding of DAMP-mediated mechanisms in tendinopathy, emphasizing their roles in inflammation, fibrosis, and tissue remodeling. We also discuss the therapeutic implications of modulating DAMP-driven signaling pathways to restore immune homeostasis and promote regenerative healing. A clearer understanding of these immunomechanical interactions provides a foundational framework for developing mechanism-based treatments that target the underlying causes of tendinopathy.

## 1 Introduction

Tendons are connective tissues that join muscles to bones, enabling force transmission and movement (1). Due to their role in transmitting high mechanical loads, tendons are susceptible to injury, particularly in athletes and individuals performing repetitive movements (2, 3). Following injury, the tendon undergoes a repair process characterized by overlapping phases of inflammation, proliferation, and remodeling (4). While unresolved inflammation can impair the remodeling process, the initial pro-inflammatory phase remains an essential component of the immune response, and premature suppression of the acute response may compromise functional tendon recovery (5).

Tendon injury triggers an acute inflammatory response characterized by the rapid recruitment of innate immune cells and the release of cytokines to clear cellular debris and initiate repair (6, 7). However, this inflammatory cascade must be strictly regulated by immune cells, which orchestrate tendon healing by dynamically modulating cytokine signaling and interacting with tenocytes (8). When immune cell polarization fails, persistent pro-inflammatory cytokine signaling drives chronic inflammation, promoting fibrotic repair and tendon dysfunction (9, 10). Notably, while acute inflammation is essential for tissue regeneration, sustained cytokine dysregulation and pathological microenvironmental changes shift the balance toward fibrosis and impaired mechanical function (11).

Although chronic inflammation in tendinopathies has long been attributed to persistent immune cell activation and cytokine dysregulation, the mechanisms underlying the sustained activation of the non-specific immune response in the absence of exogenous infectious agents remain a current research bottleneck. Endogenous danger signals (Damage-associated molecular patterns, DAMPs) released from stressed or damaged tendon matrix have been identified as key mediators of this process (12). Acting as molecular bridges that link mechanical microenvironmental changes to the immune response, DAMPs activate the innate immune system by binding to pattern recognition receptors (PRRs), thereby amplifying downstream inflammatory cascades and driving tendon degeneration (13, 14). Specifically, sustained mechanical strain drives the progressive escalation of sterile inflammation (15). In this process, alarm signals initially released by tendon cells promote the spatial recruitment of circulating myeloid cells. This process translates local mechanical strain into a system-wide chronic immune activation-like state throughout the tendon microenvironment (15). Studies have shown that DAMPs can form a positive feedback loop by enhancing leukocyte recruitment and promoting the release of pro-inflammatory cytokines by tendon cells, thus perpetuating the inflammatory response within the tendon (16). Consequently, targeted intervention against DAMPs holds potential therapeutic value for blocking chronic inflammation and promoting the restoration of tendon homeostasis.

## 2 Mechanotransduction to immune inflammation in tendinopathy

The development of tendinopathies is not merely a consequence of physical wear and tear, but rather a complex biological process triggered by abnormal mechanical loading, involving a transition from disrupted cellular mechanosensing to a pathological immune response (2, 13, 17). This cascade begins with the disturbance of mechanotransduction pathways and induces programmed matrix degradation that releases endogenous danger signals. Ultimately, these signals activate and amplifie DAMP-driven innate immune inflammation, establishing the molecular biological basis of tendon degeneration (13, 14).

### 2.1 Mechanical sensing and physiological adaptation of tendons

As a sophisticated tissue capable of withstanding high tensile loads, tendons possess a highly sensitive mechanosensing network established by resident fibroblasts through systems such as YAP/TAZ, FAK/Src and PIEZO1, enabling physiological adaptation to muscular loads (1, 17). At the upstream level of the signaling cascade, the FAK pathway is essential for mediating the mechanical coupling between the cell nucleus and the native three-dimensional extracellular matrix (ECM) and plays a critical role in regulating tendon cell migration and cell-ECM adhesion (18). Yap/Taz exerts transcriptional control over the initial mechanical response to tension loss and the subsequent activation of degradation programs in tendon fibroblasts (19). Depletion of Yap/Taz leads to a marked upregulation of matrix metalloproteinase (MMP2, MMP3, MMP13 and MMP14), thereby initiating matrix degradation (19). Under steady-state conditions, the PIEZO1 channel mediates calcium influx and activates calmodulin-dependent protein kinases by sensing shear stress generated by sliding collagen fibers sliding, thereby regulating Mkx and Scx expression and maintaining tendon development and differentiation (20–22). Studies have shown that the mechanical threshold of PIEZO1 lies near the upper limit of the physiological range, suggesting that this threshold likely acts as a limit switch for tissue deformation induced by mechanical loading (23). Excessive mechanical tensile stress can induce tendon cell apoptosis by activating Piezo1 and the downstream Calpain2/BAX/Caspase3 pathway (24).

In summary, FAK, YAP/TAZ and PIEZO1 collectively constitute a sophisticated sensing network through which tendons respond to physiological mechanical loads, maintaining cellular homeostasis under mechanical stimulation (24–28). When mechanical loads exceed the sensing threshold of this network, physiological adaptation shifts toward a pathological response: FAK signaling becomes persistently activated, YAP/TAZ is depleted, and PIEZO1 is overexpressed (19–23, 24). These three factors respectively drive the upregulation of degradative programs, relieve inhibitory constraints on matrix degradation, and initiate apoptosis (18, 24). During this process, abnormal mechanical loading upregulates lysyl oxidase (LOX) expression via the PIEZO1/YAP pathway, promoting excessive covalent cross-linking of collagen, thereby causing ECM stiffening and ectopic mineralization (25). This loss of matrix compliance further restricts physiological inter-fibrillar sliding, so that mechanical loads no longer dissipated via matrix slippage but are instead being directly converted into local shear stress on the tendon cell membranes (26). This deterioration of the physical microenvironment, together with the apoptosis induced by the three factors mentioned above (FAK, YAP/TAZ, and PIEZO1), is likely to constitute the primary driving force behind mechanically induced matrix degradation, the release of matrix-derived DAMPs, and the subsequent immune activation (18–24, 27).

### 2.2 Matrix DAMP generation and sterile inflammation cascade

As a sophisticated connective tissue dominated by collagen and organized into multi-tiered fiber bundles, tendons convert mechanical and physical signals into complex immunobiological responses when subjected to high tensile forces, through the synergistic action of integrins, the cytoskeleton and the ion channels (27, 28). Under mechanical overload, fibroblasts not only activate the MAPK signaling pathway to mediate coordinated tissue adaptation, but also initiate programmed degradation within the ECM by upregulating matrix metalloproteinases, targeting components such as collagen, hyaluronan and glycosaminoglycans (27, 28). This mechanically driven disruption of the physical matrix network forms the basis for the release of primary DAMPs.

The mechanism by which ECM fragments act as DAMPs to activate the immune system has been well documented. High-molecular-weight hyaluronan is degraded into low-molecular-weight hyaluronan fragments, which activate innate immune responses via MyD88, IL-1R-related kinase, TNFR-related factor-6, protein kinase C zeta (PKCζ) and NF-κB-dependent pathways (29). The disaccharide-linked glycoprotein biglycan stimulates IL-1β maturation and secretion in macrophages via the NLRP3 inflammasome without requiring additional co-stimulatory factors, and triggers the synergistic action of Toll-like receptor 2 (TLR2)/Toll-like receptor 4 (TLR4) and P2X7R/P2X4R on macrophage IL-1β secretion (30).

In addition to the aforementioned proteoglycans and glycosaminoglycan components, large glycoproteins in the matrix are also major drivers of sterile inflammation. For example, Tenascin-C undergoes a critical transformation in chronic or recurrent tissue injury. On the one hand, its mRNA undergoes alternative splicing to produce specific pro-inflammatory isoforms. On the other hand, post-translational modifications of its macromolecular structure expose pathogenic epitopes. These altered matrix components act as danger signals to activate the innate immune response, transforming self-limiting tissue repair into persistent and irreversible fibrosis. The continuous accumulation of DAMP subsequently triggers TLR-mediated matrix cell activation, ultimately forming a vicious cycle of fibrotic amplification (31).

Upon binding to cell surface receptors such as TLRs, these matrix-derived DAMPs rapidly transmit signals into the cell, driving a cascade of pro-inflammatory responses (30, 31). TLR-mediated activation of NF-κB leads to its translocation into the nucleus, significantly upregulating the transcriptional expression of IL-1β (30–32). Transcriptional analysis of early rotator cuff tendinopathy has revealed an increase in NF-κB signaling, including elevated expression of the serine kinase subunit IKKβ, which plays a key role in inflammation. Using CRE-mediated overexpression of IKKβ in tendon fibroblasts, researchers observed degeneration of the mouse rotator cuff tendons and the adjacent humeral head, changes associated with increased IL-1β. Furthermore, conditional knockout of IKKβ improved outcomes following surgical repair, whereas its overexpression impaired tendon healing (32).

In parallel with the activation of the NF-κB pathway, ECM fragments also induce the assembly and activation of the NLRP3 inflammasome, promoting the activation of caspase-1 and the subsequent maturation and release of IL-1β and IL-18, and intracellular ROS signaling (33). Moreover, the production of IL-1β further induces the secretion of S100 protein (34). In rotator cuff tendon injuries, inflammatory responses and ECM disorganization have also been found to be associated with upregulation of high mobility group box 1 (HMGB1) and activation of the NLRP3 inflammasome within the tendon (35).

Taken together, the data from these studies outline the complete pathological process in tendinopathies, ranging from mechanical sensing instability to programmed matrix degradation and ultimately to immune system activation (**Figure 1**). Through observations of FAK/YAP signaling defects, abnormal MMP expression, and the activation of NF-κB signaling and the inflammasome pathway, it is evident that abnormal mechanical loading is converted into a tissue-level immune response via biochemical cascades. However, the persistent pathological degeneration observed in clinical samples suggests that the tendon microenvironment does not return to homeostasis following initial injury, but rather becomes trapped in a vicious cycle maintained by endogenous danger signals. This pathological mechanism, in which ECM fragments act as DAMP sources to drive chronic inflammation, is not unique to tendons (36, 37). It has also been well documented in chronic liver disease and pulmonary fibrosis, conditions in which stress-induced hydrolysis of matrix proteins generates signaling-active matrix fragments, thereby persistently activating resident fibroblasts and innate immune cells (15, 36, 37). This suggests that sterile inflammation mediated by signal-active matrix fragments and DAMPs is a common mechanism prevalent in the fibrotic processes of multiple organs.

**Figure 1:**
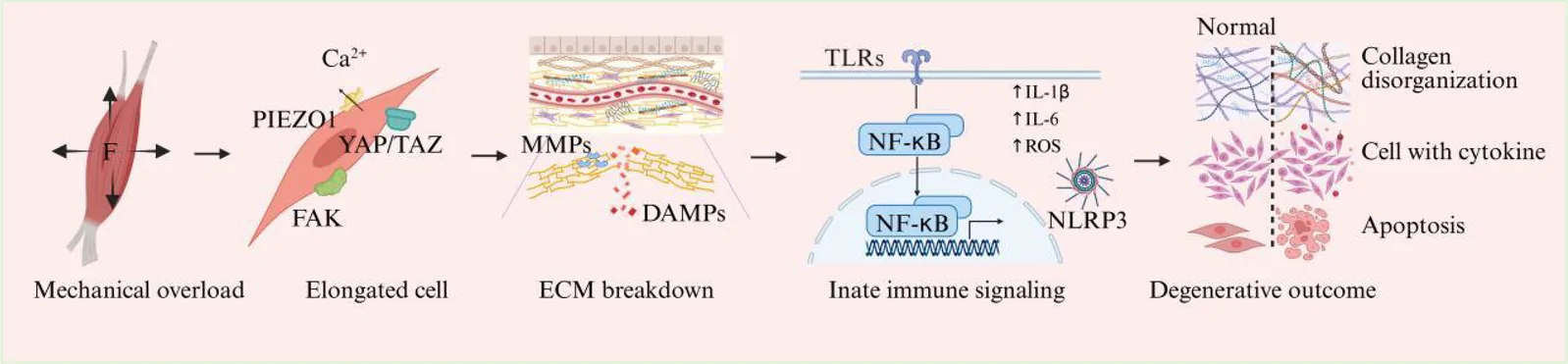
Mechanotransduction-to-inflammation axis in tendinopathy. Mechanical overload disrupts tendon mechanosensing networks mediated by FAK, YAP/TAZ and PIEZO1, triggering matrix degradation programs and extracellular matrix (ECM) breakdown. The resulting release of ECM-derived danger-associated molecular patterns (DAMPs) activates TLR/NF-κB signaling and NLRP3 inflammasome pathways, leading to the production of inflammatory mediators and oxidative stress. Sustained activation of these innate immune responses establishes a chronic inflammatory microenvironment that promotes collagen disorganization, cell apoptosis and progressive tendon degeneration (18–20, 23, 24, 27, 29–32, 35). Created in BioRender. weng, Z. (2026) https://BioRender.com/yg893om.

## 3 Key DAMPs driving tendinopathology

DAMPs derived from the ECM and those released in response to intracellular stress transform transient mechanical loads into sustained biochemical stimuli, acting as core mediators in reshaping the local immune environment of the tendon. Therefore, systematically elucidating the specific molecular types of these DAMPs and their spatiotemporal release patterns during the pathological process is crucial to understanding the unravelling logic of the progression of tendon disease from acute inflammation to chronic repair dysfunction.

### 3.1 HMGB1

Under physiological conditions, HMGB1 is primarily localized within the cell nucleus and is virtually undetectable in the ECM (38, 39). However, under conditions of mechanical stress or injury, HMGB1 translocates from the nucleus to the cytoplasm and is actively secreted into the ECM, thereby triggering subsequent inflammatory cascades (38). Clinical studies have confirmed that HMGB1 protein levels are significantly elevated in torn supraspinatus tendons compared to intact tendons (39). This HMGB1, released into the extracellular environment due to mechanical overload, is a key factor in triggering inflammatory and catabolic responses, specifically manifested as increased synthesis of prostaglandin E2 and MMP-3 in tendon cells (40).

At the molecular signaling level, extracellular HMGB1 increases the levels of phosphorylated IKKα/β, IκBα, p65, ERK and JNK in tendon cells in a dose-dependent manner, suggesting that HMGB1 primarily mediates inflammatory responses by activating the MAPK and classical NF-κB signaling pathways (41). Furthermore, extracellular HMGB1 can further induce mitochondrial dysfunction and oxidative stress in tendon stem cells (42). Alleviating oxidative stress and inflammation during tendinopathy can be achieved by HMGB1 K43 methylation, which promotes the interaction between cGAS and the mitochondrial E3 ubiquitin ligase Mul1, thereby facilitating cGAS degradation and inhibiting the activation of the NLRP3 inflammasome mediated by the cGAS-STING pathway (42).

In addition to mediating inflammatory damage in its disulfide-bond form, the biological effects of HMGB1 are highly dependent on its extracellular redox state, and it exhibits significant repair-promoting potential in a fully reduced state (**Figure 2**) (43, 44). Treatment of tendon wounds with platelet-rich plasma combined with exogenous HMGB1 reduces the CD68 marker on M1 pro-inflammatory macrophages, recruits more CD146+/CD73+ stem cells, and increases type III collagen levels, thereby promoting wound healing (43). Fully reduced HMGB1 forms a heterodimeric complex with CXCL12, binding to the CXCR4 receptor on the surface of stem cells. Through CXCR4 signaling, stem cells in the G0 phase are transformed into a pre-alert state between G0 and G1, enabling these cells to enter the cell cycle more rapidly upon receiving signals of tissue damage, thereby significantly accelerating the tissue repair process (44).

**Figure 2:**
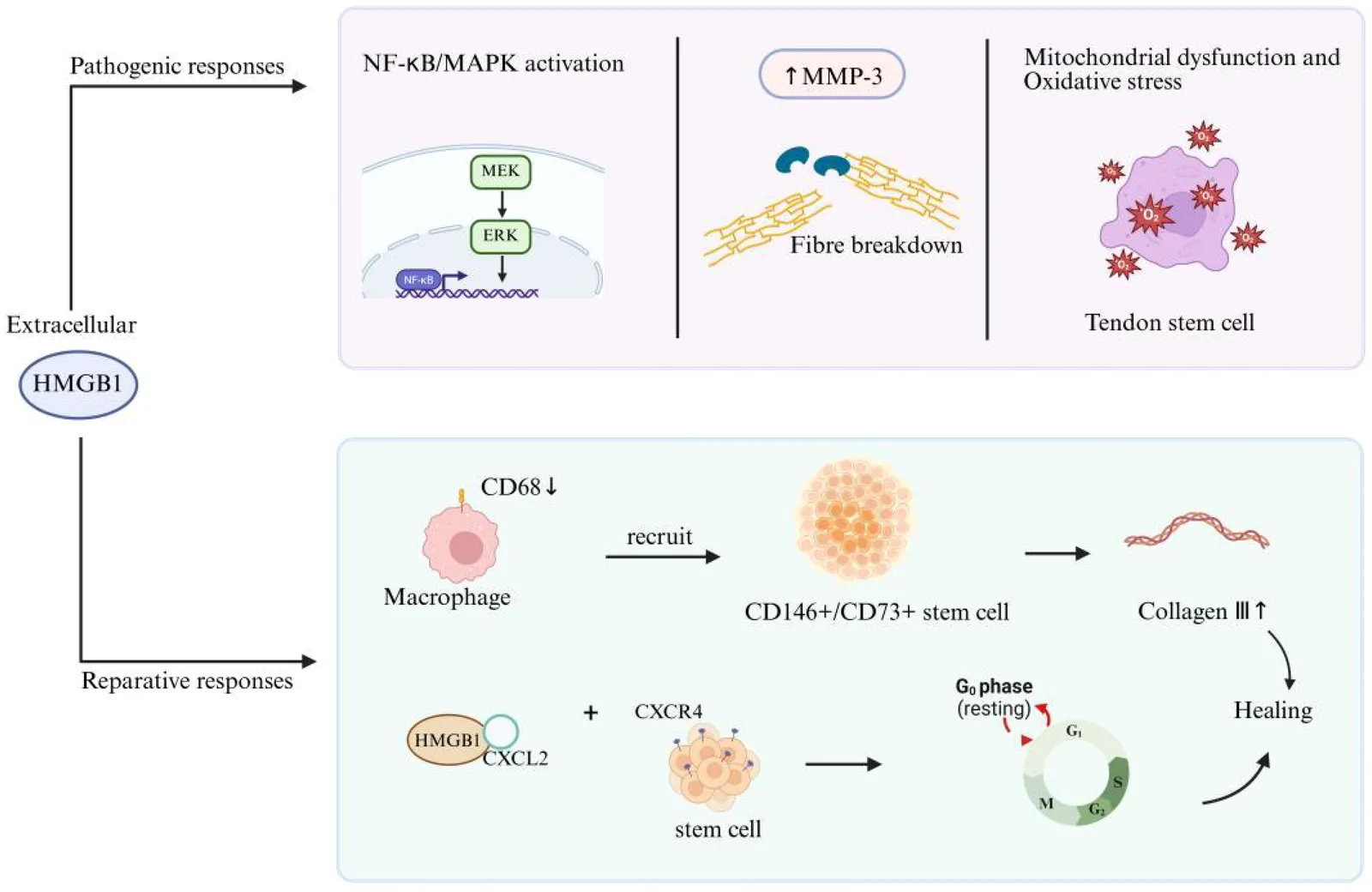
Context-dependent effects of extracellular HMGB1 in tendinopathy. Extracellular HMGB1 released from injured tendon tissue exerts both pathogenic and reparative effects. On the one hand, HMGB1 is associated with NF-κB/MAPK activation, increased MMP-3 expression, mitochondrial dysfunction and oxidative stress, thereby promoting inflammatory responses and matrix degradation. On the other hand, HMGB1 may facilitate tissue repair through CXCL12/CXCR4 signaling, stem cell activation, reduced CD68^+^ macrophage accumulation, enhanced recruitment of CD146^+^/CD73^+^ stem cells and increased type III collagen synthesis. Collectively, HMGB1 functions as a multifunctional danger-associated molecular pattern (DAMP) that contributes to both tendon degeneration and repair depending on the biological context (38, 40–44). Created in BioRender. weng, Z. (2026) https://BioRender.com/7iazx3y.

### 3.2 ATP

In the initial phase of the tendon’s mechanical response, mechanical loading induces tendon cells to release ATP (**Figure 3**) (45). As a molecule that serves both as a physiological mechanical messenger and a pathological DAMP, the biological effects of ATP depend on its extracellular concentration. Under physiological conditions, released ATP is rapidly hydrolyzed by extracellular ATPases, maintaining low concentrations and preventing cells from activating damage pathways due to an excessive response to load signals (45). When mechanical overload or cellular damage occurs, extracellular ATP concentrations may rise as ATP release exceeds the capacity of extracellular ATPases to rapidly hydrolyze it (45). Mechanical loading induces the expression of IL-1β, COX-2 and MMP-3, while concomitant autocrine and paracrine ATP release may negatively regulate these injurious responses through a feedback mechanism (46).

**Figure 3:**
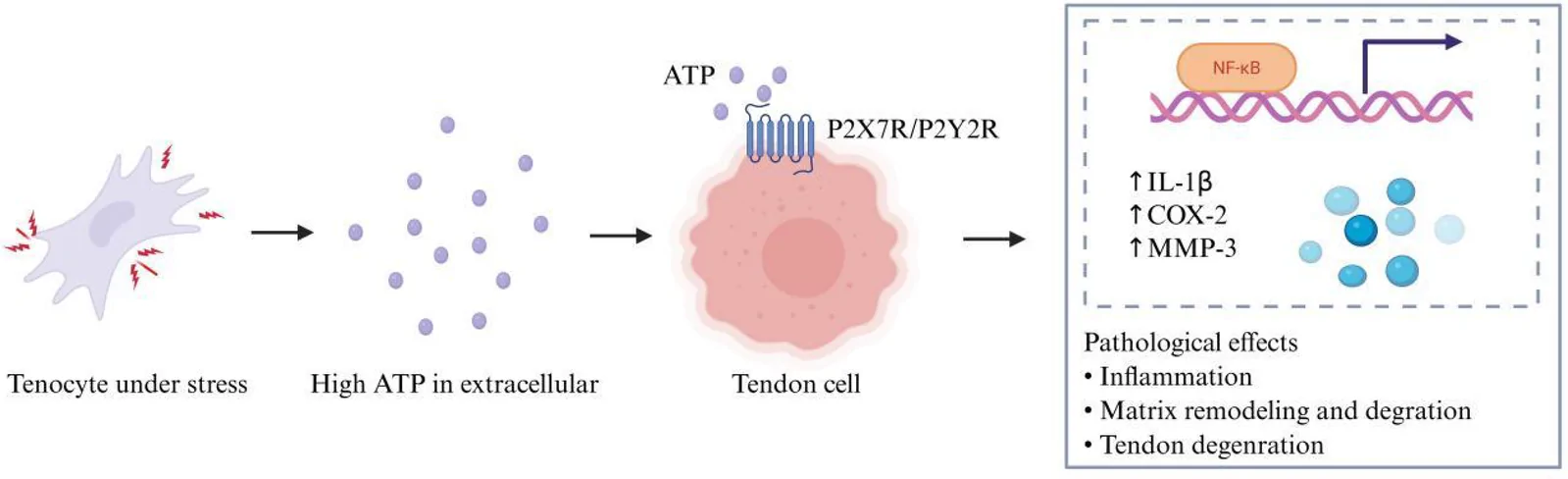
Pathogenic effects of extracellular ATP in tendinopathy. Mechanical overload stimulates ATP release from stressed tendon cells, resulting in elevated extracellular ATP levels. Extracellular ATP activates purinergic receptors such as P2X7R and P2Y2R, leading to NF-κB activation and increased expression of IL-1β, COX-2 and MMP-3. Through the amplification of inflammatory responses and matrix-degrading programs, ATP functions as a danger-associated molecular pattern (DAMP) that contributes to extracellular matrix remodeling, tendon degeneration and the progression of tendinopathy. Created in BioRender. weng, Z. (2026) https://BioRender.com/0ija2ib.

Under conditions of mechanical stress overload, tendon-derived mesenchymal stem cells of the CTSK lineage exhibit high mechanical sensitivity, primarily determining the healing or calcification fate of the tendon by regulating ATP signaling pathways (47). Targeting this regulatory node, various intervention strategies have demonstrated distinct molecular effects. High concentrations of magnesium ions can curb matrix mineralization by inhibiting the spontaneous release of ATP and the expression of P2 receptors (48). By contrast, exogenous ATP supplementation significantly reduces calcium deposition in a dose-dependent manner and improves cells survival in degenerative tendinopathy (49).

The aforementioned ATP signaling regulation and mitochondrial energy metabolism represent two distinct dimensions. Intracellular ATP deficiency resulting from mitochondrial dysfunction is a characteristic of degenerative tendinopathy but falls within the realm of energy metabolism rather than the DAMP signaling pathway. Mitochondrial transfer to damaged tendon cells via mesenchymal stem cells can effectively replenish energy, reduce cell apoptosis and modulate inflammation, thereby significantly promoting tendon healing (50). Taken together, these findings indicate that ATP signaling and mitochondrial function are interdependent, jointly constituting a key defensive axis for maintaining tendon homeostasis and preventing degenerative lesions.

### 3.3 S100A8/A9

S100A8/A9 are among the most extensively studied DAMPs in tendinopathy (16). Their pathogenic mechanisms have been systematically elucidated from multiple perspectives, including receptor recognition, cellular origin, and downstream effects. Mechanical injury first stimulates tendon cells to release CCL2, recruiting neutrophils and monocytes to the tendon microenvironment; the recruited immune cells subsequently release S100A8 and S100A9 (16). S100 proteins released into the extracellular space activate TLR4 and the receptor for advanced glycation end products (RAGE), thereby activating downstream NF-κB and MAP kinase signaling pathways (16, 51). Experimental data indicate that exogenous S100A8 and A9 induce notable increases in the protein release of IL-6, IL-8, CCL2, CCL20, and CXCL10 from primary human tendon cells. In the case of S100A9 stimulation, however, while an upregulation of MMP3 was observed, no marked changes were detected in the matrix-related genes (Col1a1, Col3a1, Decorin, Tenascin C) (16). This phenomenon suggests that S100A8 and A9 primarily influence the inflammatory features observed in tendinopathies by modulating the stromal microenvironment rather than by directly acting on matrix synthesis (**Figure 4**).

**Figure 4:**
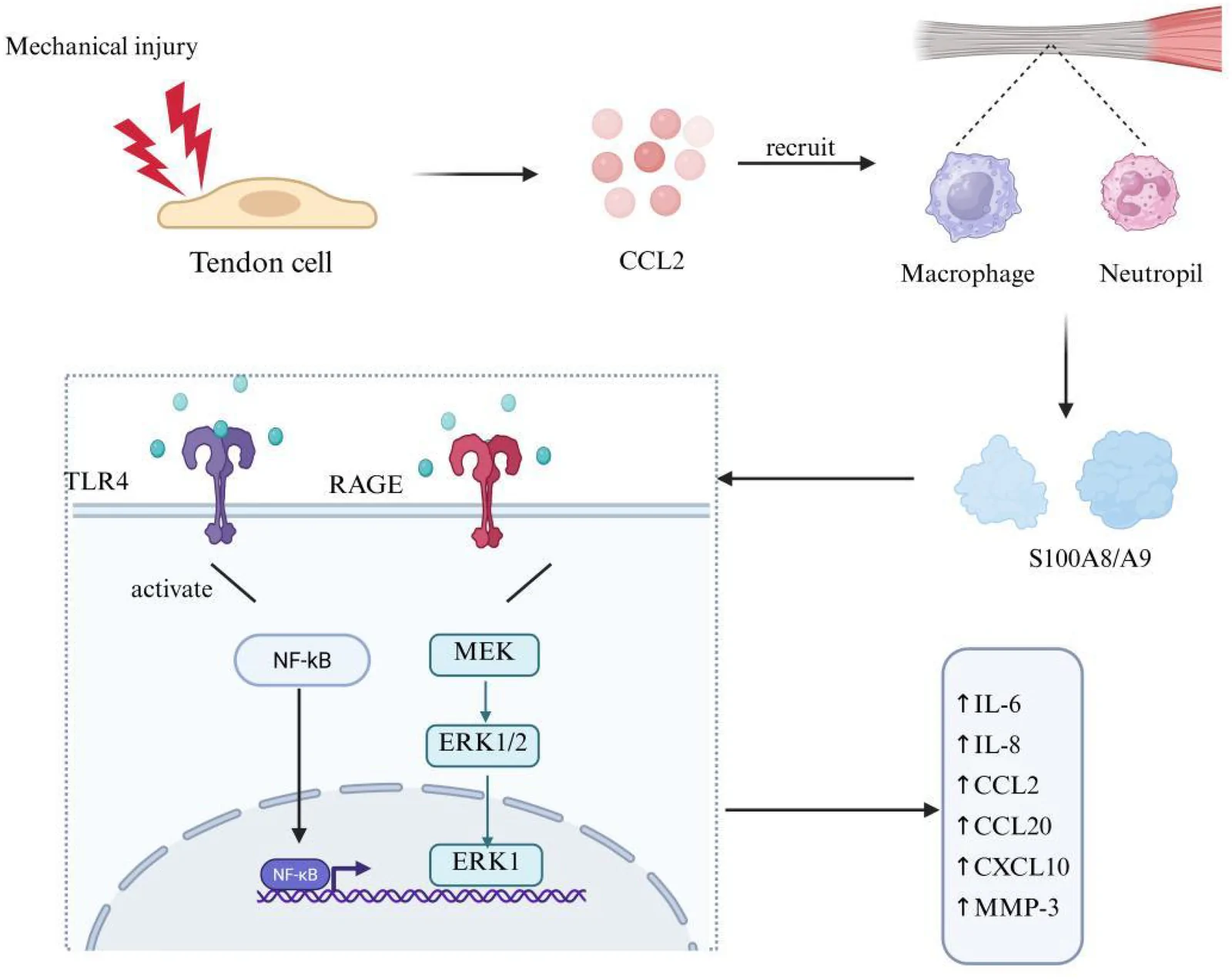
Pro-inflammatory functions of S100A8/A9 in tendinopathy. Mechanical injury induces tendon cells to release CCL2, leading to the recruitment of macrophages and neutrophils into the injured tendon. Recruited immune cells subsequently produce S100A8/A9, which act as danger-associated molecular patterns (DAMPs) and activate TLR4- and RAGE-dependent signaling pathways. Downstream activation of NF-κB and MAPK signaling promotes the expression of inflammatory cytokines and chemokines, including IL-6, IL-8, CCL2, CCL20 and CXCL10, as well as the matrix-degrading enzyme MMP-3. These responses amplify local inflammation and contribute to the progression of tendinopathy. Created in BioRender. weng, Z. (2026) https://BioRender.com/d25nqc2.

### 3.4 Other DAMPs

Various DAMP molecules are deeply involved in the progression of tendinopathies by modulating inflammatory responses, ECM remodeling and tissue repair processes. Studies have shown that mechanical stress promotes HSP47 mRNA expression and protein synthesis (52), while HSP72 is up-regulated in the nuclei of tendon fibroblasts under mechanical stress (53). During the aforementioned mechanical stress processes, HSPs can be released into the extracellular environment, where they act as DAMPs to regulate inflammatory mediators and matrix metabolism (54). Studies have found that low levels of COMP expression in tendons during the growth and development phase render horses more susceptible to tendon injury (55). Conversely, the elevated expression of COMP in tendon granulation tissue following injury further confirms its role in the tendon repair and remodeling process (56). Following tendon injury, the expression of galectin-3 is significantly upregulated and precisely localized to α-smooth muscle actin -positive fibroblasts within fibrotic areas. Targeting this site with galectin-3 inhibitors effectively reduces excessive collagen deposition in the injured area and inhibits scar formation (57). In summary, the coordinated involvement of these DAMP molecules at different stages—including inflammation initiation, structural maintenance and fibrotic repair—collectively determines the ultimate pathological outcome of tendinopathies.

## 4 Tendinopathy treatment based on DAMP signaling axis

In response to the DAMP-driven mechanostatic inflammatory cascade, the therapeutic strategies discussed in this chapter adopt a dual-targeted approach that addresses both tendon fibroblasts and immune cells. On the one hand, mechanical overload prompts tendon fibroblasts to release primary DAMPs (such as HMGB1 and ATP) to recruit and activate immune cells; on the other hand, the recruited immune cells secrete large amounts of secondary DAMPs (such as S100A8/A9), which in turn exacerbate matrix degradation and phenotypic deterioration in fibroblasts. Therefore, whether through directly intercepting DAMPs, inhibiting downstream common signaling nodes, or indirectly regulating the microenvironment, the core strategy lies in synergistically disrupting the pathological feedback loop between the two, thereby restoring tissue homeostasis.

### 4.1 Therapies directly targeting HMGB1

Although multiple DAMP molecules act synergistically in the progression of tendinopathies, existing studies on direct interventions still exhibit significant limitations in their targeting, primarily focusing on HMGB1 (38–57). Current studies in this review can be broadly categorized into two types based on drug targets: HMGB1-specific direct inhibitors and agents that indirectly regulate upstream release and the microenvironment (**Table 1**) (38, 40, 42).

**Table 1:** Therapeutic agents for tendinopathies targeting HMGB1.

Representative drug	Core molecules and cellular mechanisms	Pathological reversal of phenotype/outcome	Research stage & experimental models	Reference
Glycyrrhizic acid	Directly combine and seal the extracellular active sites of HMGB1	Block the expression of PGE2 and MMP-3 induced by exogenous HMGB1	Preclinical (*In vitro*); Primary human/rat tenocyte cultures induced by exogenous HMGB1	(40)
metformin injection	Inhibition of mechanical overload-mediated release of HMGB1 from the matrix	Reduce CD68, COX-2, and PGE2; inhibit the rounding-up of tendon cells, chondrogenesis (SOX-9, Collagen II), and protein glycation accumulation	Preclinical (*In vivo*); Intensive Treadmill Running (ITR) induced rat tendinopathy model (systemic daily injection)	(58)
metformin emulsion	Activate the activity of AMPK, and indirectly reduce the expression of TGF-β	Reduce myofibroblasts, decrease type III collagen and increase type I collagen, promoting tissue remodeling in the middle and late stages	Preclinical (*In vivo*); Tendon injury and surgical repair model (topical transdermal formulation targeting mid-to-late remodeling phase)	(59)

Glycyrrhizic acid is a representative example of a specific direct inhibitor; it can block the high-level production of PGE2 and MMP-3 in tendon cells induced by exogenous HMGB1 (40). Metformin, on the other hand, belongs to the category of indirect multi-target modulators, and its specific biological effects are precisely regulated by the route of administration. On the one hand, daily injection of metformin inhibits the release of HMGB1 into the tendon matrix induced by intensive running training, reducing the development of tendinopathy characteristics in ITR (58).

This is manifested by decreased expression of the macrophage marker CD68 and pro-inflammatory molecules (COX-2, PGE2), a shift in cell morphology from normal elongated cells to round cells, high expression levels of chondrogenic markers (SOX-9, type II collagen), and the accumulation of proteoglycans in the tendinopathic tendon (58). Conversely, the topical transdermal application of metformin emulsion focuses more on tissue remodeling in the middle to late stages of tendinopathy, mediated through increased AMPK activity, reduced TGF-β1 expression, decreased myofibroblast presence, lowered collagen III levels, and increased collagen I production (59).

Current successful interventions targeting HMGB1 provide an important clinical paradigm for targeting DAMPs signaling pathways. Several DAMPs, including S100 proteins and endogenous nucleic acids, exhibit significant functional redundancy and engage shared receptors such as TLRs and RAGE (14, 31). Given this, current HMGB1 research not only validates the feasibility of DAMPs as therapeutic targets, but also highlights the necessity of integrating multiple DAMP-interception strategies in the future to achieve comprehensive anti-inflammatory effects.

### 4.2 Therapies targeting downstream signaling nodes

Within the DAMPs signaling network, the activation of different signaling pathways often corresponds to distinct pathological stages of tendinopathy (13, 60). Firstly, the NF-κB axis, as the central hub for the initiation of inflammation, determines the intensity of the initial injury signal (60). Several pharmacological studies have demonstrated that targeting the MAPK/NF-κB pathway can effectively alleviate inflammatory responses in tendon cells and improve tissue homeostasis. For example, verapamil reduces IL-1β-induced ECM degradation and apoptosis (61);spironolactone improves tendinopathy by inhibiting inflammatory activation in tendon-derived stem cells and enhancing autophagy, thereby reducing the expression of senescence-associated proteins p16 and p53 (62). Furthermore, iridoid glycosides can downregulate the expression of inflammation- and catabolism-related molecules such as MMPs, COX-2, iNOS and IL-6, while promoting the expression of collagen I and IL-10, thereby alleviating inflammation, apoptosis and heterotopic ossification (63).

Subsequently, the JAK/STAT3 pathway plays a key role in regulating the progression of inflammation and tissue remodeling. Inhibition of JAK2/STAT3 using AG490 suppresses TGF-β1-induced fibroblast proliferation and reduces pro-inflammatory cytokines (IL-1β, TNF-α), demonstrating its therapeutic potential in preventing peritendinous adhesions (64). Aspirin suppresses inflammation and scar formation by regulating JNK/STAT-3 signaling, thereby reducing the risk of re-rupture following tendon injury (65). Connective tissue growth factors stimulates TDSCs to express IL-10 and tissue inhibitor of metalloproteinase-3 via the JAK/STAT3 pathway, suggesting that this axis promotes tendon healing while regulating inflammation (66). Under the activation of oncostatin M, early-to-mid-stage osteogenic differentiation of TDSCs is enhanced via JAK2/STAT3 signaling (67).

Finally, sustained stimulation of DAMPs signaling often converges on the NLRP3 inflammasome, driving the transition of the tendon from chronic inflammation to tissue senescence (30, 35). Inhibition of the NLRP3-ASC-caspase-1 axis via glyburide effectively reduces degeneration and calcium deposition, while promoting tendon healing (68). Celastrol, a natural triterpenoid, may inhibit angiogenesis induced by inflammatory tendon cells and promote tendon-bone healing via the NLRP3 pathway (69). Notably, the NF-κB/NLRP3 axis integrates signals from AKT phosphorylation, ROS accumulation and mitochondrial dysfunction, thereby driving senescence-associated secretory phenotype expression, while quercetin can delay senescence of tendon stem/progenitor cells senescence and promote tendon healing by attenuating this process (70). However, the overlapping signaling networks activated by different DAMPs suggest that targeting a single intracellular node may be insufficient to fully modulate the complex sterile inflammatory microenvironment (13, 14, 60). Consequently, there is a need to develop combination therapeutic strategies capable of simultaneously inhibiting multiple key nodes, or to achieve comprehensive immune regulation by targeting more upstream common mechanisms.

### 4.3 Indirect inflammatory microenvironment modulation for regeneration

Given the limitations of single-pathway targeting, current regenerative medicine strategies are shifting toward the regulation of the local inflammatory microenvironment (60). In response to the abnormal accumulation of ECM and fibrosis caused by persistent inflammation, the modulation of the pathological microenvironment at the site of injury through physical, biological or chemical means is crucial for halting the progression of tendinopathy and restoring tendon homeostasis.

In terms of physical interventions, electrical current induces an inflammatory response, promoting collagen-mediated tendon regeneration (71). In terms of biological modulations, KGN-PRP therapy effectively inhibits the activation of the AKT/PI3K/NF-κB inflammatory pathway. It significantly reduces the expression of IL-6, TNF-α and COX-2 at the tendon-bone interface, thereby lowering inflammation levels, while increasing the expression of collagen I, VEGF and HIF-1α in the regenerated tissue to promote wound healing (72). Furthermore, through precise intervention via cell-derived molecules, SHED-derived exosome-mimetics accelerate tendon-bone healing by modulating macrophage polarization, enhancing tissue regeneration, and suppressing NF-κB-mediated TNF-α/iNOS inflammation via autophagy activation (73).

Restoring mitochondrial homeostasis has emerged as a potential strategy to modulate the local inflammatory microenvironment (42, 50, 74, 75). Mechanistically, stress-induced mitochondrial dysfunction leads to excessive mtROS release into the tendon microenvironment, driving persistent tenocyte inflammation and matrix degradation. To counter this, physical interventions like blood flow restriction therapy stimulate intercellular mitochondria transfer to enhance muscle regeneration and function (74), while direct mitochondrial transplantation restores cellular bioenergetics to attenuate local tissue inflammation (75). Given the structural instability and poor localized retention of free mitochondrial therapies under physiological mechanical loading *in vivo*, injectable mechanical-adapted hydrogels provide structural support to facilitate tendon repair (76).

## 5 Conclusions and future

DAMPs occupy a pivotal position at the intersection of mechanical transmission and innate immunity in the pathogenesis of tendinopathies (12–16, 27, 31, 35). They are not merely passive by-products of tissue injury, but rather central regulatory factors in the active remodeling of the tendon microenvironment (12, 14, 31). By converting abnormal mechanical stress into sustained biochemical stimuli, DAMPs represented by HMGB1, ATP and ECM fragments induce matrix degradation, immune cell recruitment and fibroblast phenotypic transformation, constituting the logical starting point of the inflammation-degeneration vicious cycle in tendinopathy (16, 35, 40, 45, 46).

Current therapeutic explorations targeting the DAMP-PRR axis and downstream signaling pathways (such as NF-κB and NLRP3) have preliminarily demonstrated the feasibility of restoring tissue homeostasis by intercepting injury signals. However, given the heterogeneity and functional redundancy of the DAMPs spectrum, future research urgently requires substantial breakthroughs in key technologies and translational strategies.

### 5.1 Translational potential of cross-organ antifibrotic drugs

Given that specific DAMP-targeted therapies validated directly in tendons remain relatively limited, a highly promising translational strategy is to exploit anti-fibrotic drug pipelines developed for other organs. Cross-organ fibrosis arises from distinct initiating factors: for example, pulmonary fibrosis is often triggered by parenchymal cell damage, whereas tendinopathy is primarily driven by pathological mechanical overload. Despite these differences, these conditions share a conserved pathological axis: cellular degenerative damage, release of DAMPs, activation of aseptic inflammatory networks, and myofibroblast transformation (31, 36, 37, 77, 78). This shared mechanism provides a compelling rationale for repurposing anti-fibrotic drugs from other organs to treat tendinopathy.

Although these drugs have not yet been extensively validated in tendinopathy, the common molecular targets they address demonstrate the feasibility of cross-organ translation. Specifically, targeting of the Hippo pathway and hypoxia-inducible factor 1α (HIF-1α), currently in clinical development for pulmonary fibrosis, modulate matrix stiffening feedback and the hypoxic response, and are expected to block cellular phenotypic transitions induced by abnormal mechanical overload in tendons (77). Meanwhile, rosiglitazone and atorvastatin, which have shown anti-adhesion or anti-fibrotic effects in joint fibrosis, providing a rationale for further investigation in tendon injury (78). Therefore, rigorous evaluation of the biological effects of these cross-organ drugs in the tendon microenvironment will help establish new translational pathways to break the vicious pathological cycle of tendinopathy.

### 5.2 Cross-lineage cellular plasticity in tendon remodeling

Ultimately, the therapeutic success of cross-organ drugs hinges on their ability to modulate the extensive cellular rewiring within microenvironment that characterizes chronic lesions. Emerging studies indicate that the tendinopathic microenvironment involves substantial cross-lineage cellular plasticity (79–81). On one hand, chronic stress induces tendon fibroblasts to adopt a macrophage-like phenotype governed by transcriptional regulators such as c-Myc, MafB, PU.1, and C/EBPα, thereby endowing local stromal cells with an active role in driving inflammation and matrix degradation (79, 80). On the other hand, infiltrating macrophages are highly sensitive to fibroblast-derived TGF-β, which triggers a macrophage-to-myofibroblast transition (MMT) via the Smad3 pathway, exacerbating scar formation (81).

This bidirectional feedback loop underscores the need for extreme temporal precision in therapeutic intervention. To effectively guide cellular plasticity toward functional reversal rather than pathological scarring, quantitatively defining the physiological versus pathological thresholds of DAMPs is of critical clinical significance. Future research should utilize advanced coupled mechanobiological-biochemical models to delineate the precise concentration profiles and kinetic windows that mark the transition of DAMPs from physiological adaptation to pathological decompensation. Building upon these threshold windows, drug development efforts should focus on engineering multivalent macromolecular scavengers or stage-specific controlled-release delivery systems tailored to specific pathological phases (82, 83). Such strategies will enable the precise, localized remodeling of the tendon immunomechanical ecosystem, ultimately translating both cross-organ drug candidates and cellular reprogramming insights into successful clinical therapies.
